# Single‐cell spatial transcriptomics reveals shared transcriptional responses to amyloid proximity in Alzheimer's disease and type 2 diabetes

**DOI:** 10.1002/alz70855_098987

**Published:** 2025-12-23

**Authors:** Roy Lardenoije, Angela R. S. Kruse, Lukasz G. Migas, Claire F. Scott, Cody Marshall, Morad C. Malek, Adel Eskaros, Thai Pham, Kristie Aamodt, Madeline Colley, Melissa A. Farrow, Raf Van de Plas, Alvin C. Powers, Matthew Schrag, Jeffrey M. Spraggins, Joana P. Gonçalves

**Affiliations:** ^1^ Delft University of Technology, Delft, Zuid‐Holland, Netherlands; ^2^ Vanderbilt University, Nashville, TN, USA; ^3^ Vanderbilt University Medical Center, Nashville, TN, USA; ^4^ Vanderbilt University School of Medicine, Nashville, TN, USA; ^5^ Veteran Affairs Tennessee Valley Healthcare System, Nashville, TN, USA; ^6^ Vanderbilt Brain Institute, Nashville, TN, USA

## Abstract

**Background:**

The presence of amyloid pathology can have a profound effect on the surrounding cellular neighborhood. While this impact has been mainly investigated for amyloid plaques in the context of Alzheimer's disease (AD), other forms of amyloid deposits can also be found in the brain and in other organs. In the pancreas, amyloid deposits consist of islet amyloid polypeptide (IAPP) and are a hallmark of type 2 diabetes (T2D). Notably, T2D has been associated with an increased risk of developing AD, and as such T2D is a common comorbidity of AD. It has therefore been suggested that these diseases may share pathophysiological processes. To advance our understanding in this respect, we compared the cellular and transcriptomic responses related to the proximity of amyloid pathology across the AD brain and T2D pancreas.

**Method:**

Xenium single‐cell spatial transcriptomic profiling was applied to tissue sections from a human post‐mortem AD brain (150,060 cells) and a T2D pancreas (256,907 cells). Spatial transcriptomics images were integrated with amyloid histopathology images to determine the proximity of individual cells to amyloid deposits. Together with cell type predictions, this enabled the investigation and cross‐organ comparison of amyloid‐associated changes in cell type composition and gene expression changes.

**Result:**

With respect to cell type composition, in the brain a higher proportion of microglia could be observed close to amyloid pathology, while in the pancreas this was mirrored by a higher proportion of macrophages as well as a higher proportion of activated stellate cells. Cell type specific differential gene expression analysis based on amyloid proximity revealed many cell types with altered gene expression, including astrocytes, microglia, oligodendrocytes and endothelial cells in the brain and acinar, alpha and activated stellate cells in the pancreas. Comparison across organs revealed 16 shared genes differentially expressed with proximity to amyloid deposits, including *CAV1, CXCR4, MS4A6A, SNCG*, and *SOX2*.

**Conclusion:**

Here we spatially investigate the impact of amyloid deposits on the cellular and transcriptomic microenvironment in the brain and pancreas. Our analysis revealed a common set of amyloid proximity related genes, providing insight into potentially shared pathological pathways underlying AD and T2D.

